# The biorepository portal toolkit: an honest brokered, modular service oriented software tool set for biospecimen-driven translational research

**DOI:** 10.1186/s12864-016-2797-9

**Published:** 2016-08-18

**Authors:** Alex S. Felmeister, Aaron J. Masino, Tyler J. Rivera, Adam C. Resnick, Jeffrey W. Pennington

**Affiliations:** 1 Department of Biomedical and Health Informatics, The Children’s Hospital of Philadelphia, 3401 Civic Center Blvd, Philadelphia, PA USA; 2 College of Computing and Informatics, Drexel University, 3141 Chestnut Street, Philadelphia, PA USA; 3 Department of Neurosurgery, Perelman School of Medicine at the University of Pennsylvania, 3400 Civic Center Boulevard, Building 421, Philadelphia, PA USA

**Keywords:** Biorepository research, Translational bioinformatics, Precision medicine, Honest broker, Cancer genomics, Data integration, Data representation, Open source, Patient health information protection, Patient privacy

## Abstract

**Background:**

High throughput molecular sequencing and increased biospecimen variety have introduced significant informatics challenges for research biorepository infrastructures. We applied a modular system integration approach to develop an operational biorepository management system. This method enables aggregation of the clinical, specimen and genomic data collected for biorepository resources.

**Methods:**

We introduce an electronic Honest Broker (eHB) and Biorepository Portal (BRP) open source project that, in tandem, allow for data integration while protecting patient privacy. This modular approach allows data and specimens to be associated with a biorepository subject at any time point asynchronously. This lowers the bar to develop new research projects based on scientific merit without institutional review for a proposal.

**Results:**

By facilitating the automated de-identification of specimen and associated clinical and genomic data we create a future proofed specimen set that can withstand new workflows and be connected to new associated information over time. Thus facilitating collaborative advanced genomic and tissue research.

**Conclusions:**

As of Janurary of 2016 there are 23 unique protocols/patient cohorts being managed in the Biorepository Portal (BRP). There are over 4000 unique subject records in the electronic honest broker (eHB), over 30,000 specimens accessioned and 8 institutions participating in various biobanking activities using this tool kit. We specifically set out to build rich annotation of biospecimens with longitudinal clinical data; BRP/REDCap integration for multi-institutional repositories; EMR integration; further annotated specimens with genomic data specific to a domain; build application hooks for experiments at the specimen level integrated with analytic software; while protecting privacy per the Office of Civil Rights (OCR) and HIPAA.

## Background

Current research is yielding rapid advances in personalized, precision medicine through targeted therapies based on an individual’s genome, genomic biomarkers, and cell biology across adult and pediatric translational research [1, 2]. This type of research has become increasingly dependent on the collection of large cohorts of high quality human biospecimens that are paired with clinical annotations [3]. While biospecimen-driven research is widely practiced, it is often limited in scope because it requires time-consuming manual processes such as retrospective annotation, cohort identification and institutional human subjects research oversight [4]. Consequently, many academic medical centers are creating large institutional biospecimen resources that can be leveraged by numerous investigators [5]. There is a trend towards these resources becoming indispensable in academic medical centers [3, 6].

Biorepository data is typically captured in longitudinal, asynchronous workflows that pose software design and data integration challenges [7]. An optimal system must provide de-identified, granular and longitudinal data to researchers while also enabling data collection workflows that require patient identification [8]. The required data often resides in separate systems such as a Laboratory Information Management System (LIMS), Research Data Capture tools, the Electronic Health Record (EHR), genomic data stores and high performance computing clusters [9, 10]. Integrative solutions are necessary at the point of collection and at information and specimen retrieval. The data must be curated to ensure it is persisted in an understandable representation for researchers in a specific medical domain [11, 12]. As biorepository resources include more clinical information and grow in scale, there are more opportunities for protected health information (PHI) to be injected into the process [13, 14]. Therefore, a central component to this toolkit is an informatics-based approach to honest brokering [15]. We build on methods described in Dhir et al. and Boyd et al. that describe specific implementations of software to aid in the honest brokering between various types of clinical data collection and de-identified biorepositories [14, 16]. We take a slightly different approach by creating non-user facing software *service* similar to Boyd, et al. for the honest broker as one of many components of a toolkit of connected operational biorespository informatics resources. We remove the human component completely from the de-identification and re-identification of research records in connected research systems.

In this paper, we address the creation of a robust biorepository management platform that enables association of a physical biospecimens, clinical diagnoses, and patient, genomic and research. This platform utilizes a modular software architecture developed at the Children’s Hospital of Philadelphia (CHOP) in partnership with the Children’s Brain Tumor Tissue Consortium (CBTTC) [17]. The platform was developed in the specific context of distributed biorepository and biobank studies in biological tissue and genomic research. In this manuscript, we describe the requirements, challenges in architectural design and implementation to create integrated data resources in biospecimen-driven translational research. We designed and utilized an open source, modular software toolkit that supports biorepository operations and de-identified secondary usage. We created an operational and scientific resource that protects subject privacy, allows for variable specimen and data management workflows and flexible resource queries. When new systems and workflows are introduced, the toolkit allows for flexible introduction of new data types, systems and operational workflows spanning specimen, clinical, imaging and genomic data. Our platform allows for extensible software and data resources for biospecimen-driven translational research.

### Cancer focus

Cancer is a main focus of current precision medicine initiatives. This is reflected in politics, the media, public funding and medical research community priorities [18]. We are in an age of increased use of web technologies that allow us to reach new levels of productivity and connectivity in business, finance, government and entertainment [19]. The time has come for us to use these same techniques to unravel the complexities of cancer [20]. New breakthroughs are helping us use our own immune systems to target an increasing list of common cancers [21]. Unfortunetely, time is not on the side of children suffering from rare brain tumors. Recent research and government population health programs identify over 120 types of pediatric brain cancers [22]. To make matters more complex, the origins of brain tumors in kids is widely unknown [22–25]. Pediatric cancer patients are treated for cancers with adult-based therapies and there is a lack of investment from pharmaceutical companies in the specific diseases affecting children [26]. It is essential to create biologic- and data-centric resources to find pathways and molecularly describe disease seen in research similar to Bastianos et al. and Parsons et al. where developments, respectively, uncover a molecular pathway in Craniopharyngiomas and a comprehensive molecular description of the common childhood brain tumor, Medulloblastoma [27, 28]. Though molecularly based research has become common with the availability of high throughput technologies, further progress is needed in infrastructure, specifically in cancer research, that allows for complete clinical annotation of specimen and genomic data from consented subjects ([29], p. 549).

Initial research of rare tumors at CHOP brought biorepository data sharing, management, and annotation issues to the forefront. A need for enhanced capabilities was particularly evident in two proposed studies targeted by our initial software system design. These tumor biorepository studies originally used a human honest broker to manage the de-identification and re-identification of records to exclude protected patient information (a/k/a patient health information) from the research LIMS. The process began with manual data intake by a data manager on hardcopy REDCap case report forms (CRFs) [30]. The CRFs contained the patient identifiers: Medical Record Number (MRN), First Name, Last Name and Date of Birth. The CRF was physically delivered to a human honest broker that would create a new electronic REDCap record with a research identifier. The hardcopy CRF was then returned to the data manager with the research identifier attached and the patient identifiers removed. The data manager then abstracted the hand written CRFs to the associated REDCap project record. Each longitudinal data collection event required manual re-identification by the human honest broker. This process became unsustainable as biospecimen and clinical data collection increased and molecular experimentation associated with records began. It was also difficult to complete the CRF in a single patient encounter due to variations in encounter length and frequency. This experience clearly illustrated the need for a scalable solution that would abide by NCI Best Practices for Biospecimen Resources [15]. The Biorepository Portal Toolkit (BRP Toolkit) project was subsequently developed to support biorepository development at institutional scale.

## Methods

### Modular approach

We took a modular and entity-driven integrated systems approach to facilitate variable specimen acquisition and data collection events. The primary entity is the patient enrolled as a research subject on the study. The subject entity is created in the electronic honest broker (eHB) and assoicated with a master patient index (MPI) and the subject’s instutional origin. Each external research record associated with the subject record, in this case the data management tool, REDCap, is not limited to a one-to-one relation of subject-to-REDCap record. The subject entity can be assoicated with many projects, forms and records in a 1-to-many entity relationaship [31]. We, in tandem, built a research portal, dubbed the Biorepository Portal (BRP). The BRP can access subject records in the electronic Honest Broker (eHB) and subsequent external research records through token-based authorization from that client system. The BRP reproduces the REDCap electronic Case Report Forms (eCRFs) based on records stored in the eHB with a custom REDCap client utilizing the REDCap application programing interface (API), in real-time. This produces a complete form for that subject at time of access. It displays the subject information and identifiers at the top of the screen at all times during form data entry and while shifting from form to form. A research coordinator or data manager can enter any temporal and longitudinal research data based on their protocol subject list at anytime or in any order (i.e., asynchronously) while maintaining the continuous de-identification and re-identification of research data automatically.

The CHOP Biorepository Core Facility utilizes ThermoFisher Nautilus as its LIMS. As part of our method, we also built a client to this LIMS that allows for association of an arbitrary number of specimen records in the LIMS with the corresponding subject record. In this way specimens can also be collected longitudinally over time. Data and specimen coordinators have the ability to associate sets of specimens with a subject or event and annotate that specimen on the fly in one system. For downstream integration, we use the same eHB software service to perform our Extract Transform Load (ETL) processes that are tailored to each project. The result is a regularly updated non-human subject research database that allows for seamless queries spanning research and clinical data sets. We allow collaborators to access specific sets of data via the data exploration tool, Harvest [32], customized for each project. The phenotypic data associated with specimen records can be integrated with direct clinical data from the EHR with appropriate institutional permissions. The modular approach allows us to integrate genomic visualization tools at the specimen level where applicable. For cancer genomics specifically, we utilize the CBioPortal [33, 34], an open source tool to visualize mutation and gene expression data from The Cancer Genome Atlas (TCGA).

### Web service oriented architecture

**Fig. 1:**
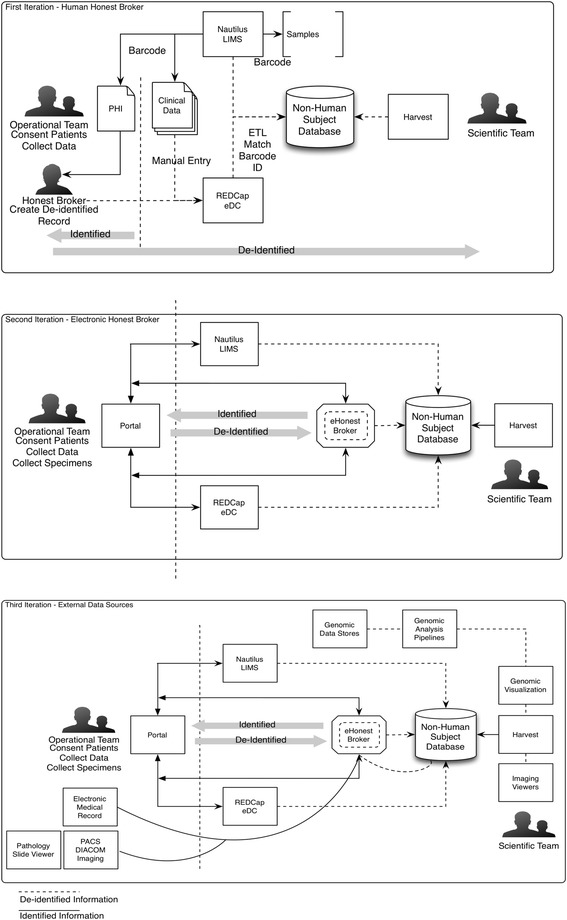
Three workflow iterations. Iterations of the introduction of the modular tool kit from human intervention, electronic intervention to fully realized modular approach

The integration of tools is accomplished by taking advantage of modern web technologies. Our methods are rooted in web service oriented architecture (SOA). This pattern pervades the current generation of computing and web technology and is rapidly expanding through virtual resources accessed via network resources (i.e., cloud computing) [35]. We created a plug-and-play experience working with multiple tools in a web environment. We employ REST (Representational State Transfer) API architecture over HTTP protocols providing uniform channels for applications integrated into the tool kit [36]. In this section, we describe each specific technology in the stack, each with its own set of RESTful end-points that allow us to guide users through multiple tools as they interact with biorepository resources. Though SOAs are widely used in the field of biomedical informatics to build complex application tool chains, they are not transparent as to their usage and sometimes have very poor adoption because of their complexity [37]. To address this, we mask technical complexity with end-user tools that are familiar to research coordinators, specimen coordinators and data managers. There is wide recognition that there is no compliant Health Level Seven (HL7)-type interoperability standards when using SOA’s [9]. The operational applications and scientific applications we implemented utilize an honest broker software service, a well-known method of dealing with de-identification in biorepository research that maintain the HIPAA compliance in downstream systems [14, 38–41]. Figure 1 illustrates a high-level architecture description along with external integration points for key scientific and clinically relevant data points for data entry and reuse. This figure is split into three parts, the last of which describes the fully realized modular approach integrating both clinical, genomic and research specimen resources.

#### Electronic Honest Broker (eHB) software service

The concept of an honest broker has been implemented in other academic medical center environments to protect privacy when integrating research data ([14, 42], pp. 56–107). Central to our solution is the eHB, a web-based software service with end-to-end encryption that maintains an index of subjects linked to their associated research records. The initial studies/projects targeted with this solution began as a manual paper-based process of considerable complexity, and are now an “informatics tool” [15]. The index in the eHB uniquely identifies each patient through a combination of organizational association (e.g., The Children’s Hospital of Philadelphia) and a unique organization provided identifier (e.g., Medical Record Number). The eHB associates each subject record with trusted external systems and known record identifiers in those systems. For annotated biorepository studies, the eHB maintains associations to de-identified records in REDCap and the LIMS. Following our SOA approach, the eHB makes data and system functionality available via a REST web service. This allows the addition of new data management application clients in a way that is system- and programming language-agnostic. To control access, the eHB uses token-based authorization, and encrypts its data both at rest and in transit, relying on client-side keys to decrypt the payload received from the API. For known applications, the eHB provides subject data with few restrictions. Client applications determine the context of what information is appropriate to display to a user, thereby enabling flexibility to meet different workflow and protocol requirements.

**Fig. 2:**
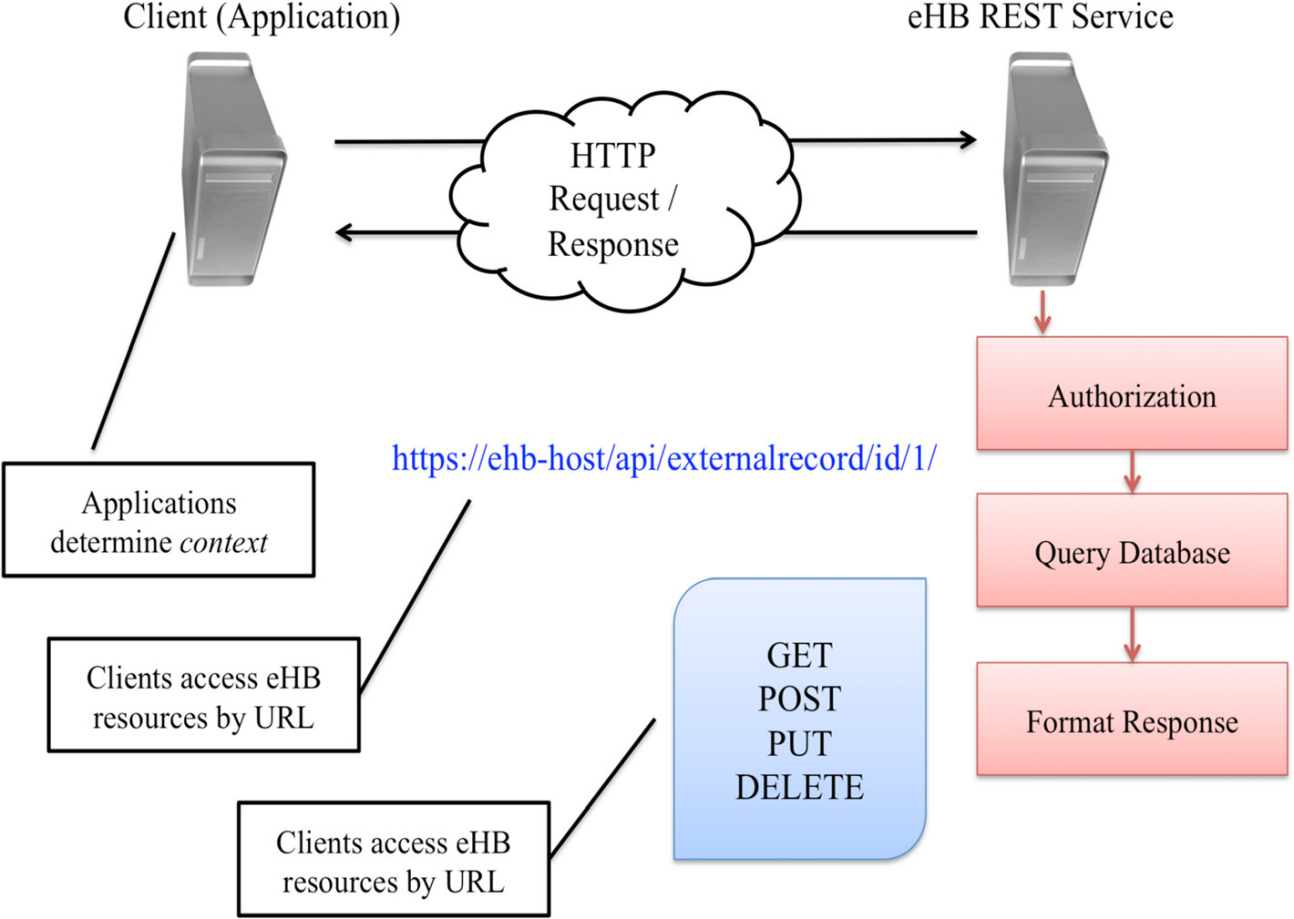
The interaction between the eHB and client applications. This takes place over HTTP through CRUD (GET, POST, PUT, DELETE) operations. Client applications (i.e., the BRP) determine the context of the information sent and received to the eHB service. Within the eHB, data is encrypted at rest, and in transit. A query of data can only take place through the eHB software service

The eHB has a limited web-facing user interface that allows for the administration of access tokens and users. It can be managed through a comprehensive set of create, read, update, delete (CRUD) operations exposed by the REST API. Client applications, described in subsequent sections, determine the context of the request. The client application requests resources of the eHB service via a URL endpoint secured using transport layer security (TLS) and, with appropriate keys and credentials, can read and write data to and from the eHB service. The eHB REST service handles authorization of the application, encrypts data and formats a response. The actual database behind the eHB service stores only encrypted information and would be unreadable if accessed. This type of encryption decouples the identifiers and encrypts any and all information going into the eHB database and can be considered “privacy-by-design” by selectively sending and granting access to information based on context and only storing the minimum set of information needed to stitch together a record for data management or data query [16, 43]. The architectural design of the eHB, illustrated in Fig. 2, utilizes web request type architecture to be a completely independent component of the tool-kit. The eHB model is similar to prior research in clinical informatics and the EHR. Architecturally, the health record must have the element of being future-proof. There is an assurance of openness and portability through standards, flexibility and scalability, semantic interoperability and acceptance from the domain experts. In Blobel et al., the authors discuss the fundamentals of future-proof health systems describing an “atomic component” [44]. We apply this notion by creating an “atomic component” that must be guaranteed utilizing the eHB to associate the subject on the study (as the atomic component) with their biospecimens, phenotypic and genomic data.

#### The Biorepository Portal (BRP)

**Fig. 3:**
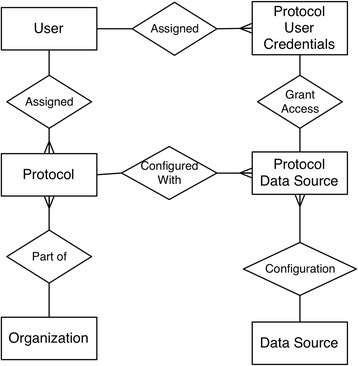
Sudo ER diagram of complex relationships between users and data sources. Users are granted access to subject records based on protocols. Each protocol groups a list of subjects that only authorized users can manage and are part are assigned to. Within the walls of a protocol, there are groups of subjects that allow for users to differentiate between institutional or site-based cohorts (i.e., organizations). Each unique subject enrollment creates a new record for that subject in the eHB. Within a protocol there are specific data entry points set up as protocol data sources (e.g., a REDCap case report form project or LIMS specimen project), and each user has credentials to each data source. Data sources are set up as clients to each external system represented in the BRP. In our case, we have created data sources for two systems, REDCap and ThermoFisher Nautilus LIMS

The eHB service discussed in the previous section is exposed solely to external client systems through APIs and lacks a user interface. The user-facing BRP component provides an integrated view of multiple data management software applications. We implement a web-based application that uses HTTP protocols to communicate with the eHB and external systems. The BRP allows research staff to work with subject identifiers, subject research data and associate specimen records. The BRP presents the honest brokered data by integrating custom clients using external system APIs to integrate in real-time, the research data and patient identifiers and records. The BRP allows access via authentication utilizing institutional identity management systems that comply with all network access guidelines for hospital clinical systems then limits users to specific cohorts, institutions and data resources accessed through the portal. Figure 3 describes the layers of user access to protocols and data sources representing applications connected to the tool in a sudo-ER diagram.

**Fig. 4:**
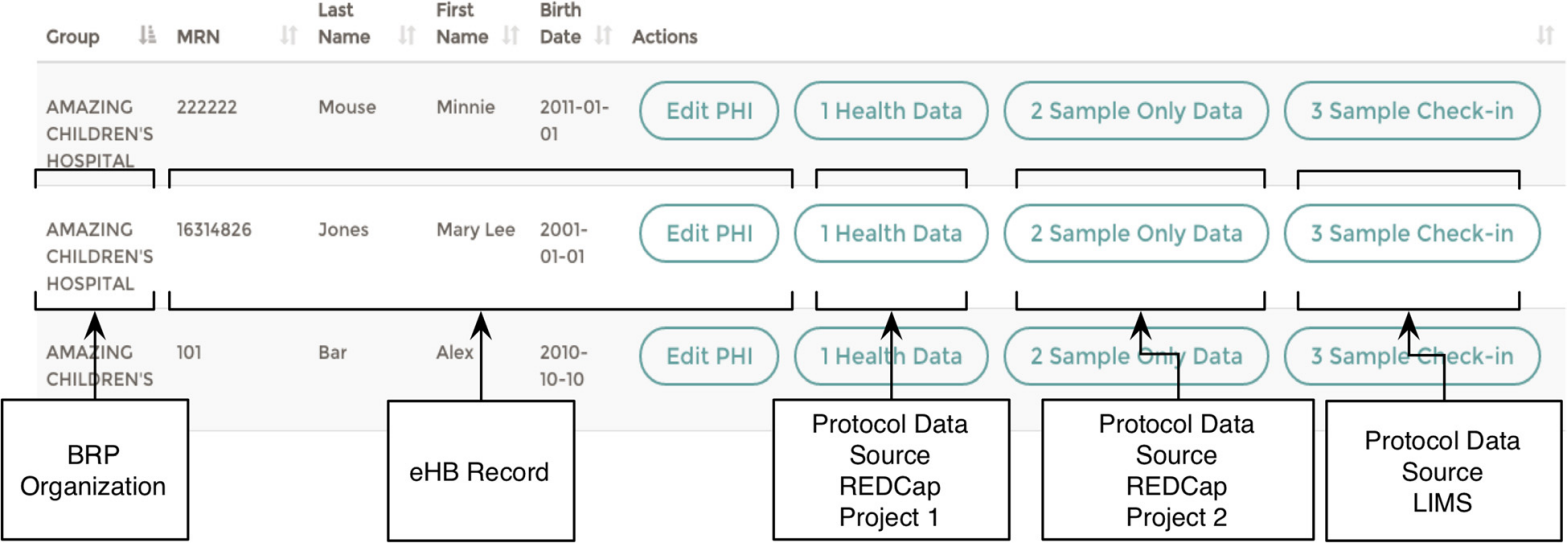
Screenshot example. A screenshot from the BRP displaying identifiable data entry along the de-identified entry points for client systems’ data sources

The BRP provides context to operational tools through clients to tools used in the context of biospecimen management. These clients are configured in the BRP as *data sources*. Display options for each client can be specified by a configuration encoded in JSON [45]. JSON formatted configuration files enable the portal to manage the display and access of REDCap forms and events in the data management processes based on workflows for capturing longitudinal data. The BRP not only relates an honest brokered subject record to external research systems, but also relates records to each other across systems. The following describes two implementations of external data sources; in this case a laboratory system and a data management system. In this case, records in these systems are linked in the BRP to provide further context around the connection of external research system (i.e., specimen collection record to case report form). Figure 4 is an example of the record listing in the BRP.

### Data sources

#### REDCap client

The REDCap client in the BRP makes a request for the metadata and data from a subject record stored in the eHB and recreates the form requested utilizing the REDCap API. The REDCap form client shows the patient Medical Record Number (MRN), last name, first name and date of birth along with each customized research data capture form. After the form record is saved, the BRP utilizes the eHB software service to either create a new record or modify an existing record in the REDCap project. The REDCap project record identifier is hashed and randomly generated without use of derived patient information. eHB identifiers are generated utilizing the application client key, in combination with a salted hash value which is guaranteed to be unique [46]. Creating a research identifier not derived from a direct patient identifier is required when using patient data for research [13]. Research identifiers are created by the connected research system randomly and are not derived from any patient information.

Adding a layer that removes the subject entity from the REDCap projects associated with a study allows for REDCap to facilitate user access to projects, form building, data logging, and managing a study data dictionary [30]. The ability to supplement an entire REDCap project(s) or form(s) as specimen annotations is accomplished by associating another REDCap project with a portal project and, in turn, a subject. Our approach includes the ability to have variable numbers of projects and nested project records per patient. There are many variations of studies that use a variable number of REDCap projects/forms and project records depending on the need of the domain. For example, a BRP protocol can capture demographics one time in one REDCap project while collecting many diagnosis-type forms with longitudinal events in another project that allows for multiple records per subject. The eHB mediates and stores the links between the subject entity and their project records. Conversely, we allow for the tools to maintain separate cohorts of identified subjects where the data are stored in the same REDCap project. This is particularly important for studies in which multiple institutions are participating in sending data and specimens to one data and specimen-coordinating center (DCC/SCC). The link to REDCap records depends on the domain and temporal requirements of a biorepository study.

#### LIMS client

A key requirement in our choice of a LIMS was that it implements an API that exposed the ability to create new specimen records, print labels and update specimen records with tags from external systems. The LIMS assigns a unique identifier to a specimen collection event, and this identifier is associated with the subject entity in the eHB by user input via the BRP. The BRP has a custom client that allows specimen coordinators with the proper credentials to associate pre-labeled specimen accession kits with the subject entity. Specimen collection kits with proper collection tubes and labels are created prior to subject enrollment in the CHOP Biorepository Core Facility.

The specimen coordinator then scans the barcoded label on the kit through a LIMS client in the BRP to associate the kit with the patient. Any downstream laboratory work, for example; receiving, processing, analysis and storage are performed directly in the LIMS. The laboratory technicians processing and receiving specimen kits do not see patient identifiers, only the LIMS assigned identifier. This facilitates the longitudinal capture of multiple specimen collection events associated with one subject.

### Access

#### Extract transform load

As Goble, et al., describe service oriented technology mechanisms “…[o]nce plumbed, the data have to be massaged and cleaned to make them fit together or conform to new schema” ([36], p. 689). We meet this requirement with ETL scripts written to integrate the disparate and de-identified data together for scientific use by researchers. This ETL process acts as another application with client access to the eHB. The first part of the ETL script uses application key-driven access to obtain a list of subject entities on specific protocols linked their respective research identifiers in data management applications. This linked list is used throughout the ETL process to join together and integrate data from disparate research systems and perform further de-identification where required by a study protocol. The ETL process produces data in a relational domain model suitable for researcher query. The ETL process is also where we integrate systems and data that are not part of the data entry in the BRP. If allowed by the protocol, the ETL process can query the eHB for patient records and pull clinical data from the health record and move it to the non-human subject biorepository database.

#### Researcher query tool and non-human subject research data resource

**Fig. 5:**
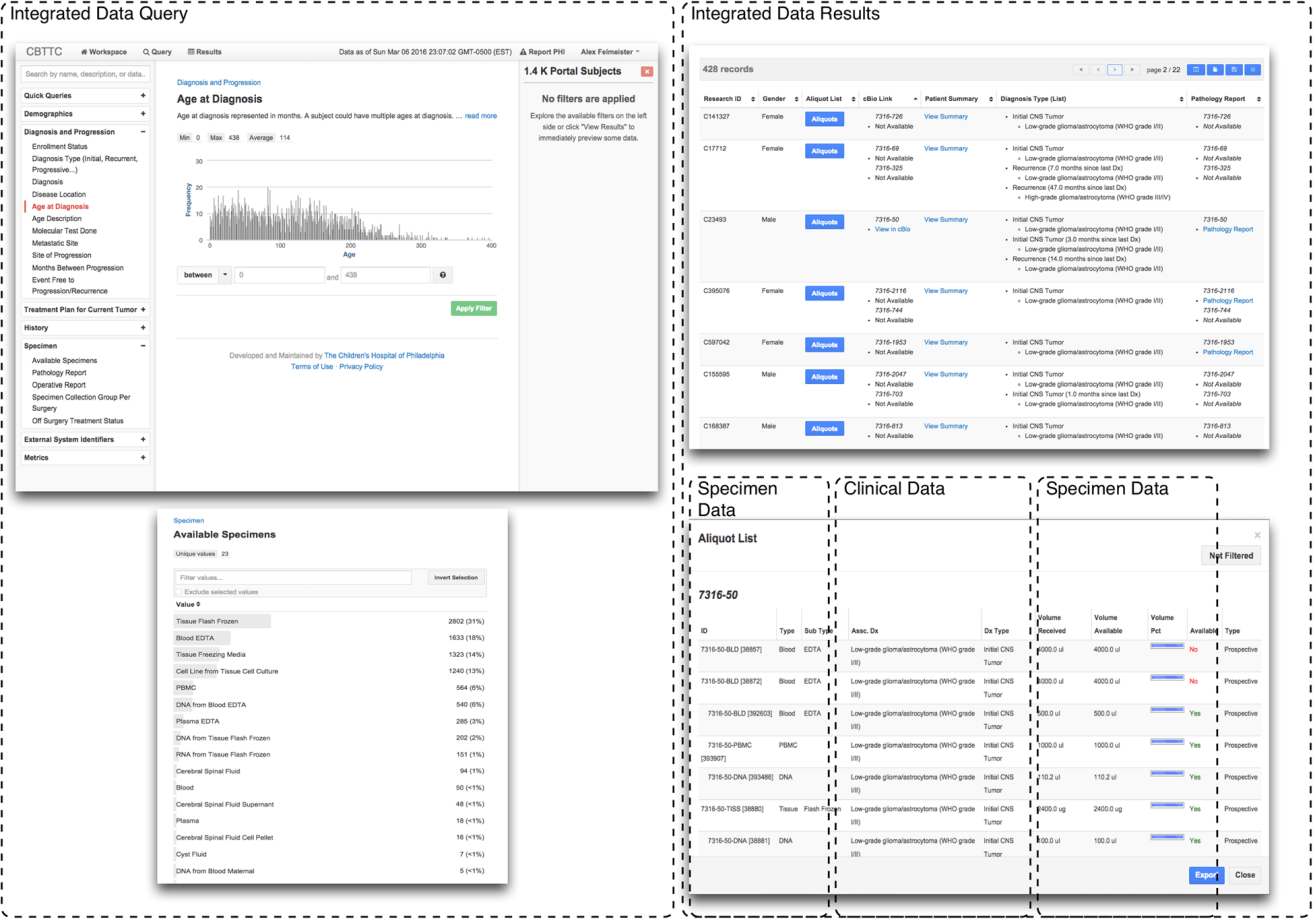
Integrated researcher view. Integrated non-human subject data and specimen query tool built on the Harvest platform. This interface allows for query across multiple systems in one place. The platform can be customized to allow for only certain data elements to be utilized that depend on domain and researcher requirements. Exposed in this example are elements captures in CRFs and the LIMS. There are also links out to genomics analysis tools

Integrating data sets through an ETL process is the starting point to fully realize the research potential of an integrated biobank. Researchers need to be able to discover available data and formulate queries for case definition and cohort creation. To enable this, we created a query tool that allows researchers to get quick answers to questions using available data without involving the Institutional Review Board (IRB) because all data is de-identified in the query tool [11]. This tool is implemented using the open-source Harvest framework [32]. Harvest gives the informatics team the ability to customize the application where necessary, but also have an out-of-box query tool for domains of relevant data. An example of a Harvest instance where users can search the multi-institutional biospecimen and annotation data of the CBTTC is shown in Fig. 5. This figure shows point-and-click access to multi-dimensional and disparate data in one place.

## Results and discussions

### Usage results

**Fig. 6:**
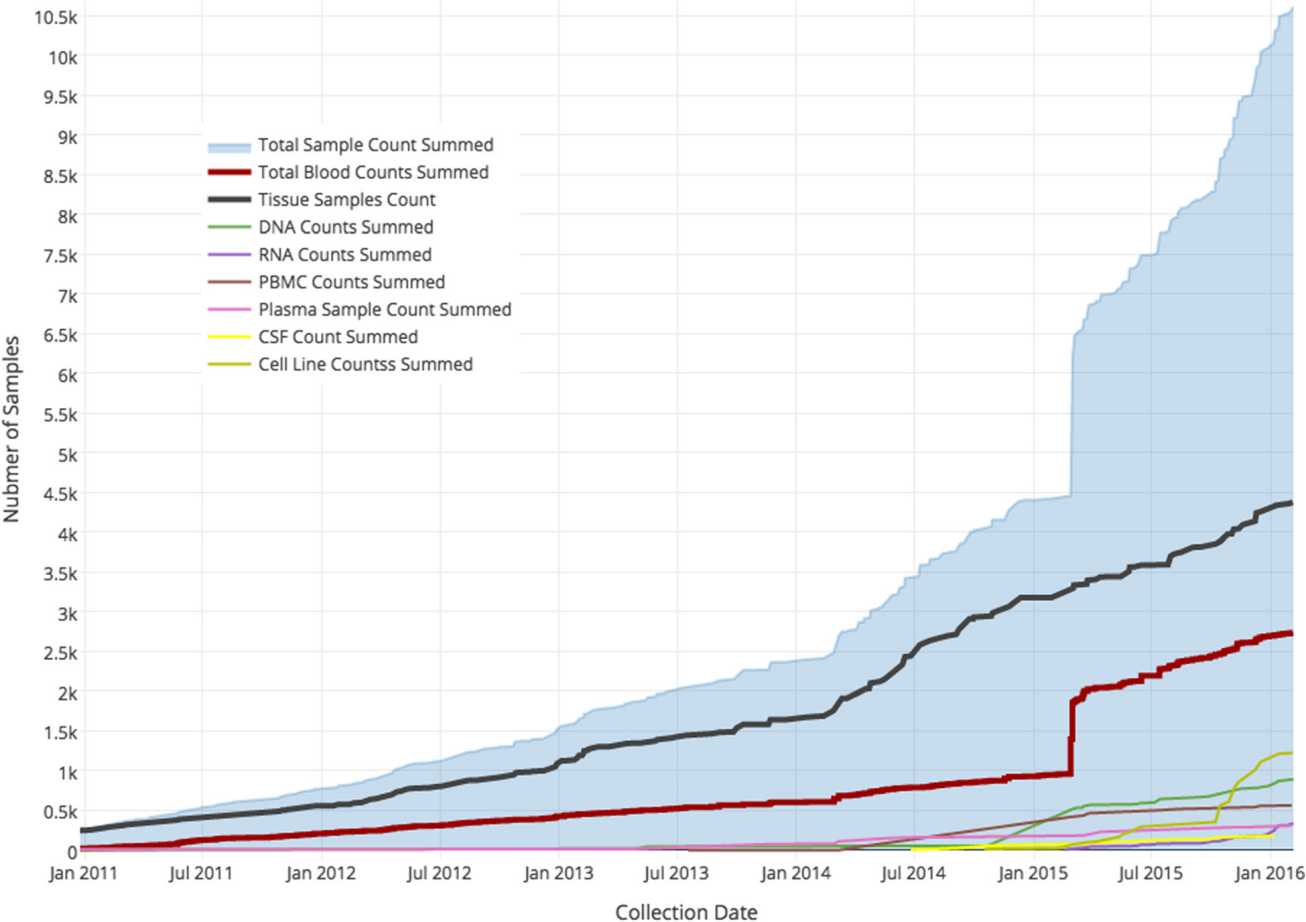
Usage. A graph of specimen accessioning for the CBTTC which has accessioned over 10,000 specimens to date (end of January 2016). The blue shaded area represents the sum of all specimens collected by the project

The toolkit supports multiple biospecimen-driven research studies. In these studies, the accessioning of specimens and related data has grown and completely changed in scale and volume. Figure 6 is a graph showing specimen accessioning 2011 to January, 2016 for the CBTTC. In 2012 the toolkit was adopted and the graph illustrates the change in accession events happening after the toolkit adoption with a varied pace of specimen accession. This means data is available to researchers in near real-time as accessioning happens compared to pre 2012 where data was only available as the operations center could sort and enter data. Table 1 is a list of projects that utilize the toolkit with summary counts of subjects, specimens and scientific data points as of the end of 2015.

**Table 1:** Project list this table is a list of select high-volume projects utilizing the modular tool-kit architecture described in this manuscript that particularly integrate multiple research resources through the toolkit

Project	Subjects (rounded)	Specimens accessioned (rounded)	Data integration points
The Children’s Brain Tumor Tissue Consortium	1400	9200	-Case Report Forms
			-LIMS
			-Cancer Genomics
University of Pennsylvania-CHOP Neurosurgery Tissue Collaborative	1500	14,000	-Case Report Forms
			-LIMS
IBD Center Biorepository Studies	177	1100	-Case Report Forms
			-LIMS
			-Electronic Medical Record
Center for Childhood Cancer Research	470	2200	-Case Report Forms
			-LIMS
			-Electronic Medical Record
PennCHOP Microbiome Center	60	1500	-Case Report Forms
			-LIMS
			-Electronic Medical Record
			-Genomic Analysis Pipelines

The table includes the project, a rounded number of individual subjects consented to the study, specimens and the integrated data points for the project. These numbers are rounded and are from the end of year, 2015

### Operational and scientific complexity

Our methods allow for a significant amount of complexity in scientific data management. With a modern, web-service oriented architecture we abstract the subject entity from multiple related project data in supporting research systems, providing increased flexibility and adaptability over comparable monolithic systems. The tools facilitate the longitudinal collection of clinical phenotypic data over an arbitrary time period. We also promote asynchronous and variable collection of specimens.

### Protection of subject privacy

The architecture of the eHB helps to protect patient privacy by limiting exposure of patient identifiers to research staff. Identifiers are only available to study operations staff responsible for associating data and specimens with subjects in the context of specific IRB protocols. The toolkit shares implementation of security and access controls with the connected downstream research system (e.g., access and logging features of REDCap). Similarly, authorization to connect LIMS specimen records to a patient record are configured with LIMS named users, and the toolkit relies on the security protocols exposed through the LIMS API. Researchers only access secondary data resources that result from downstream ETL and are never directly connected to the honest broker component of the tool kit. The ability to query the eHB allows the team to build domain specific data repositories and a web query tool limited risk of exposing patient information.

### Evolving data types and workflows

Downstream data management systems and workflows can change project to project or as the science changes within a project. We allow all downstream applications do what they were designed to do without any impact on the underlying architecture of the toolkit. The toolkit is resilient to change, and the service-oriented architecture provides hooks to incorporate additional systems, reflecting the certainty of changing requirements, data and workflows.

### Discussion

Annotation of biospecimens with longitudinal clinical data was the initial impetus of creating this toolkit, but it has gone much further. The BRP and REDCap integration have enabled multiple multi-institutional biorepository studies, both international and national. As in Harris et al., we enable concurrent multi-institutional projects to be controlled by staff through a single common interface similar to other new research informatics initiatives [30]. Exploration of HL7 for Oncology and honest brokering is ongoing, but we have not been able to implement these medical informatics type interchange languages for this purpose [47]. Additionally, web services were not built to be HIPAA compliant [9], but we find that designing modular tools that have privacy built in by their specific usage to be sufficient in research. Further, we find the modern web architecture to allow for the integration of the favored web tools scientists are using for their research to be a novel approach.

### The Children’s Brain Tumor Tissue Consortium

In Fig. 5, the data and specimen query tool of the Children’s Brain and Tumor Tissue Consortium, is some of the results of ambitious biorepository collaboration between six children’s hospitals. Informatics staff utilize this set of integrated tools to support the data and specimen-coordinating To date, the toolkit has been central to a number of grant-funded projects focused on next generation sequencing of biospecimens, and the identification of causative mutations in tumors and data integration heavy microbiologic research (See Table 1). A researcher can quickly determine study feasibility by reviewing available specimens and data in childhood brain cancer research and formulate new studies focused on finding pathways in rare cancer similar to Brastianos et al. and Parsons et al. [27, 28]. Current efforts are focused on integration of highly dimensional genomic data sets generated by such studies.

### EMR integration

Integrating or retrieving data from the Electronic Medical Record (EMR) is a growing need for users of the toolkit. Projects originating at CHOP have been able to take advantage of the eHB to re-identify patients for the purpose of extracting clinical data from the CHOP EMR. We are able to, with appropriate IRB permission, obtain and de-identify clinical data directly from the EMR and incorporate these data as annotations on specimens and genomic data. This has proven useful in studies that require observational data such as medication at time of specimen collection, particularly in high frequency specimen collection in microbiologic research.

### Genomic data integration

**Fig. 7:**
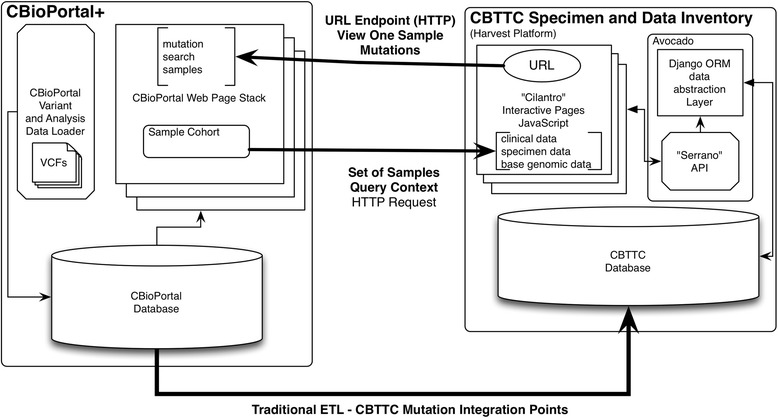
CbioPortal – harvest integration architecture. The integrated query tool allows for a back and forth search between genes of interest and visualization in CBioPortal, and the tool for phenotype and specimen requests. We perform this integration in a similar fashion to the other tools in the tool-kit. We utilize a combination of constructing web endpoints and traditional ETL. The cancer genomics integration starts with a scripted pull of mutation data via a secure database connection utilizing elements of CBioPortal’s relational data structure to store this large set of data. Specimens known to the repository are loaded into the CBioPortal by the bioinformatics team with known specimen identifiers from the LIMS. This creates a natural link between any granular genomic data, the specimen and ultimately the subject. URLs are constructed in the query platform that allow for researchers to move from clinical and specimen driven queries directly to CBioPortal to visualize mutation data of interest

**Fig. 8:**
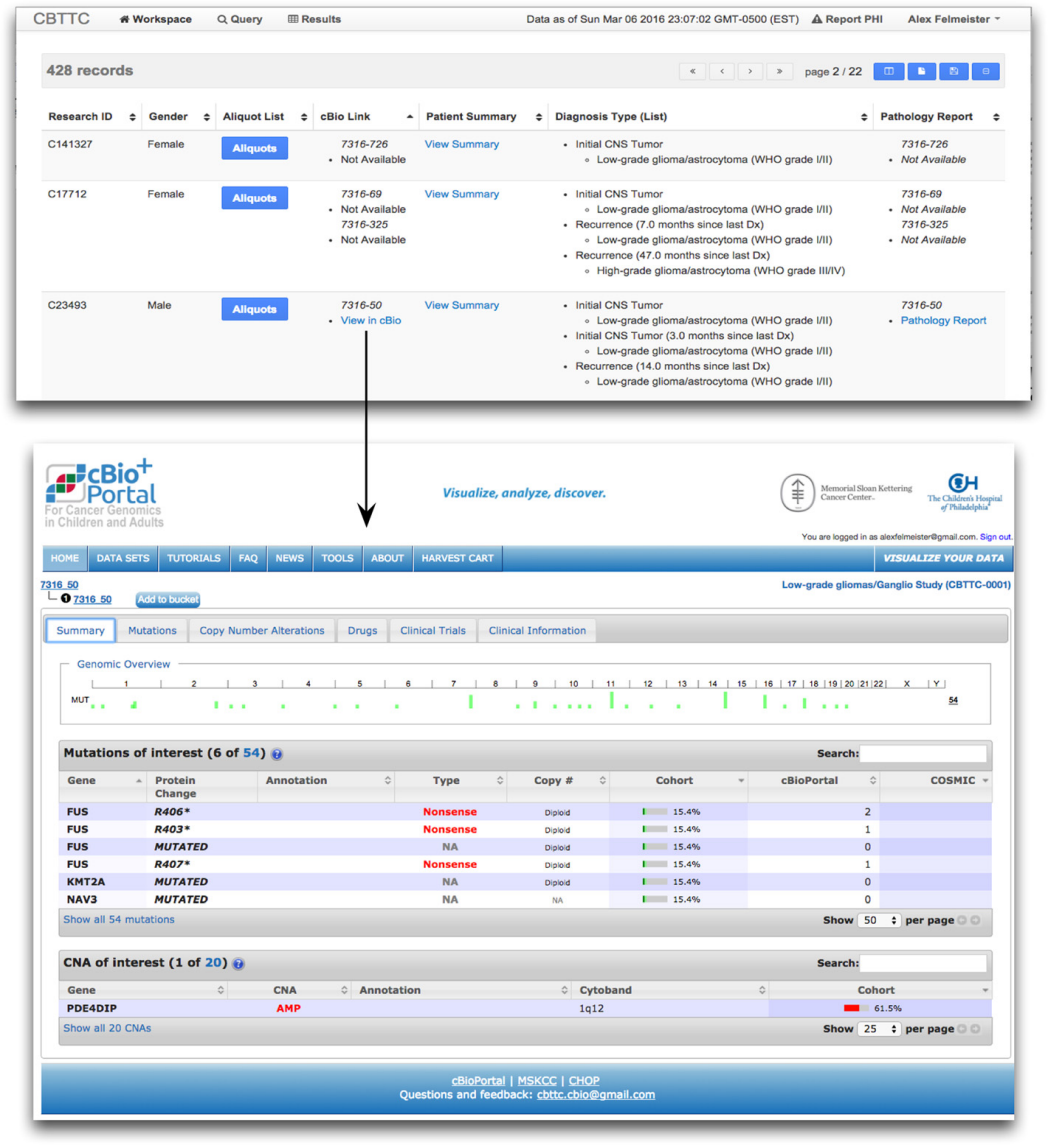
Specimen to cancer genomics. Screen shot of integration of the Harvest-based data and specimen query tool with a the CBioPortal for cancer genomic visualization of a specific case

Continued success of biorepository-driven research relies on tight integration of other scientific data points. A primary example of this is the phenotypic and genomic information relation. In the case of the cancer tissue biorepository, the specific cancer genomic alterations are specifically important to annotate specimens. We found that allowing a researcher to query specific somatic mutations from comparative analysis of sequencing the germ-line and the tumor and find the physical specimens with that mutation to be a powerful feature. We accomplish the query of specific tumor mutations by integrating the CBioPortal into our toolkit. The CBioPortal an open source tool developed to view cancer-based genomic analysis data originally developed at Memorial Sloan Kettering Cancer Center (MSKCC) is an open access resource for exploration of multi-dimensional cancer genomics data sets [33]. Figure 7 shows how the integration of the CBioPortal with the clinical and specimen based query tool with a mix of traditional ETL and web based endpoints. Figure 8 is a set of screen shots of this cancer genomic data integration. This integration point opens up a capability unavailable in previous tissue focused biorepositories. Users can search for and view mutations called on specimens in the local biorepository data repository to pull a physical specimen for biological research. Conversely, the user can search for pathways of interest across published studies and discover if those pathways are shown in local specimens. The user can be moved back to the specimen request tool with the context of specimens from a genomic mutation-based query. Similarly as we continue to add scientific platforms to the scientific side of the toolkit pertinent to domains we are integrating with multiple commercial data management service providers. We will extend this concept by pushing the platform further to incorporate workflows dedicated to the classification of pathology diagnoses and collaborative discussion about pathology calls derived from high-resolution pathology slide scans. All of this would not be possible without a modular approach.

## Conclusions

As of January of 2016 there are 23 unique protocols/patient cohorts being managed in the Biorepository Portal (BRP). There are over 4000 unique subject records in the electronic honest broker (eHB), over 30,000 specimens accessioned and 8 institutions participating in various biobanking activities using this tool kit. We specifically set out to build rich annotation of biospecimens with longitudinal clinical data; BRP/REDCap integration for multi-institutional repositories; EMR integration; further annotated specimens with genomic data specific to a domain; build application hooks for experiments at the specimen level integrated with analytic software; while protecting privacy per the Office of Civil Rights (OCR) and HIPAA. To meet this challenge, we created an open source modular software toolkit that automates many manual components of biorepository data workflows while also protecting patient privacy. Conversely the modular solutions allow for novel integration points for translational research spanning clinical and genomic data. We believe this work advances the state of the art within the biomedical domain by moving towards modern technologies and architectures to provide translational research resources.

## Availability of software

*Project Name*: The Biorepository Portal Toolkit

*Project home page*: http://www.brptoolkit.com

*Code Repositories*:

- Electronic Honest Broker Service: https://github.com/chop-dbhi/ehb-service
- Electronic Honest Broker Client: https://github.com/chop-dbhi/ehb-client
- Biorepository Portal: https://github.com/chop-dbhi/biorepo-portal
- Data Sources: https://github.com/chop-dbhi/ehb-datasources

*Operating Systems*: Linux

*Other Requirements*: Docker (optional but recommended)

*License*: We believe in open source software and open-source our work. The Biorepository Toolkit and all integration work with non-proprietary systems is licensed under BSD 2-clause License.

*Restrictions to use by non-academics*: None.

*Ethics approval*: No ethics approval was required for this work.

A demonstration of this software is available at http://www.brptoolkit.com. This website also contains documentation, webinars, descriptions and pointers to code repositories in context. Software discussed in this paper is available on the CHOP Department of Biomedical and Health Informatics github repository at https://github.com/chop-dbhi. A specific implementation of the toolkit is available through the Children’s Brain Tumor Tissue Consortium at http://www.cbttc.org.
